# A Metabolites’ Interplay Can Modulate DNA Repair by Homologous Recombination

**DOI:** 10.3390/ijms27031517

**Published:** 2026-02-03

**Authors:** Valentina Rossi, Mirco Masi, Marzia Govoni, Marina Veronesi, Martina Duca, Stefania Girotto, Andrea Cavalli, Giuseppina Di Stefano

**Affiliations:** 1 Department of Medical and Surgical Sciences (DIMEC), Section of General Pathology, University of Bologna, 40126 Bologna, Italy; valentina.rossi78@unibo.it (V.R.); martina.duca3@unibo.it (M.D.); 2 Computational and Chemical Biology, Italian Institute of Technology, 16152 Genoa, Italy; mirco.masi@iit.it (M.M.); andrea.cavalli@iit.it (A.C.); 3 Centre for Applied Biomedical Research (CRBA), University of Bologna, 40126 Bologna, Italy; marzia.govoni@unibo.it; 4 Structural Biophysics Facility, Italian Institute of Technology, 16163 Genoa, Italy; marina.veronesi@iit.it (M.V.); stefania.girotto@iit.it (S.G.); 5 Department of Pharmacy and Biotechnology, University of Bologna, 40126 Bologna, Italy; 6 Centre Européen de Calcul Atomique et Moléculaire (CECAM), Ecole Polytechnique Fédérale de Lausanne (EPFL), 1015 Lausanne, Switzerland

**Keywords:** lactate, butyrate, cancer cells, DNA repair, homologous recombination

## Abstract

Small molecules either derived from cell metabolic reactions or produced by gut bacterial flora have shown the potential of affecting gene expression, which suggests the possibility of interactions able to modulate cellular functions. In this context, the reported experiments were aimed at verifying a possible interplay between lactate and butyrate in modulating the efficacy of antineoplastic drugs. Butyrate is a product of gut bacterial flora, shown to be endowed with anticancer properties; conversely, increased lactate levels in cancer cells were found to be associated with higher proliferation and drug resistance. For the reported experiments, we adopted two cell lines from clinically relevant, but different cancer forms: pancreatic and triple-negative mammary adenocarcinomas. In spite of their different tissue origin, the two cell lines appeared to similarly respond to the effects of the two metabolites, which were found to modulate in opposite ways the expression of key genes involved in DNA repair by homologous recombination. As a consequence, changed efficacy of this repair pathway and modified response to PARP inhibitors were observed. Notably, our results also suggest that the counteracting effect between these two metabolites may be leveraged to address additional challenges limiting the success of anticancer therapies.

## 1. Introduction

Ever increasing evidence suggests that cell metabolic pathways can give rise to small molecules endowed with regulatory properties on gene expression [1,2]. The modulation of cellular functions operated by these metabolites is believed to be useful in helping adaptation to changes in microenvironment, resulting in improved cellular functions.

In this context, some recent studies by our research group contributed to highlight the role of lactate in working as a signaling molecule in cancer cells [3,4,5,6]. As is well known, neoplastic change is characterized by highly upregulated glucose uptake and glycolysis, resulting in substantially increased lactate production (the so-called Warburg effect) [7]. This metabolite was found to induce histones’ hyperacetylation and/or lactylation, two epigenetic changes leading to increased DNA transcription [8,9]. We showed that the enhanced lactate levels which characterize neoplastic tissues can be involved in the reduced efficacy of commonly used antineoplastic agents [5,6], also favoring the onset of drug resistance [3,4]. Coherently with the role of the upregulated glycolytic metabolism observed in embryonic tissues, in cancer cells this metabolite was also found to promote cell proliferation and infiltrative growth [10].

It can be hypothesized that the cell-intrinsic regulatory mechanism linked to metabolic reactions could be modified by the interaction with molecular species of external origin, such as those derived from the individual microbiota. The possibility that microbial interactions linked to dietary habits could impact carcinogenesis and the therapeutic response of cancer cells is an actively debated question, not only related to neoplasms arising in the digestive tract [11].

Butyrate is one of the main metabolites derived from gut bacterial fermentation and has been recognized to play a major role in the microbiota-associated anticancer benefits [12]. The molecular mechanisms involved in the antineoplastic effects of butyrate are different, but they can be mainly attributed to histone deacetylases (HDAC) inhibition [13]. The increased histone acetylation induced by butyrate was found to be correlated with cell cycle arrest (caused by the reduced expression of the cell cycle-related genes) and with apoptosis induction (linked to the reduced expression of the bcl-2 family genes) [14,15]. Interestingly, this molecule was also shown to impact cell metabolic regulation [16]. In butyrate-exposed cancer cells, improved mitochondrial function and oxidative metabolism were observed; these effects were found to be linked to the inhibition of glucose transporter-1 expression [17] and to the activated hexokinase activity [18]. Additionally, in HeLa and HepG2 cells butyrate was found to cause sirtuin-3 inhibition, leading to activation of the pyruvate dehydrogenase complex and to reversion of the Warburg effect [19].

These observations led us to explore whether the modulation of gene expression induced by this metabolite could potentially counteract some of the epigenetic effects linked to the increased lactate levels characterizing cancer cells, which could give a further mechanistic explanation of the anticancer effects of butyrate.

To test this hypothesis, we used the following two human neoplastic cell lines, representative of clinically relevant tumor forms: the pancreatic adenocarcinoma BxPC-3 and the triple-negative breast adenocarcinoma MDA-MB-231. In previous experiments, we had already observed that in BxPC-3 cells the expression of some proteins involved the homologous recombination pathway that was related to LDH activity [20]; in addition, a study performed on MDA-MB-231 cells highlighted that increased lactate levels can lead to EGFR pathway activation via HB-EGF, and can promote infiltrative growth [6].

## 2. Results

### 2.1. Butyrate Reduces Lactate Production and Histone-3 Lactylation in Both BxPC-3 and MDA-MB-231 Cells

**Figure 1:**
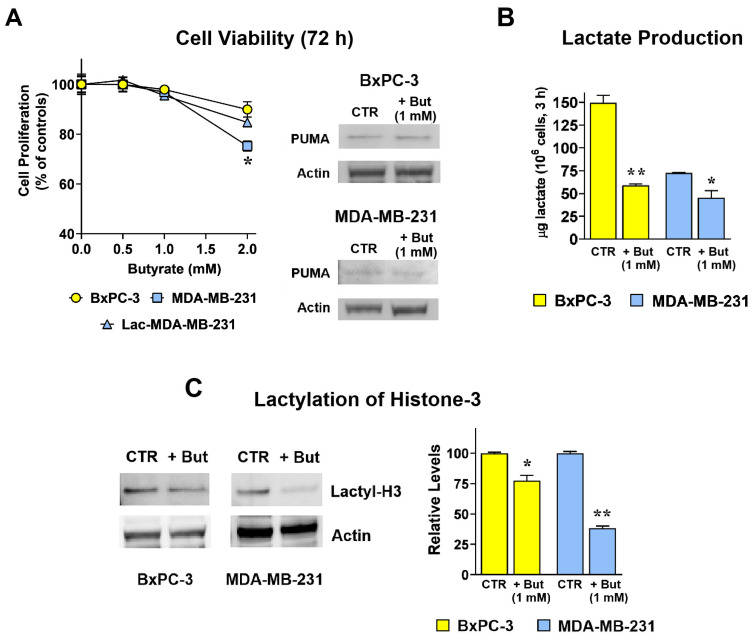
Butyrate reduces lactate production and histone-3 lactylation without affecting cell viability. (**A**): Effect of butyrate on cell viability. No statistically significant effect was observed at 72 h for doses ≤ 1 mM. The 2 mM dose caused a ≈20% reduced cell proliferation in MDA-MB-231 cells, with a statistically significant difference vs. the lactate pre-conditioned cells (Lac-MDA-MB-231; *p* < 0.05, as assessed by Student’s *t*-test). The tolerability of 1 mM butyrate (+But) for the cell lines was confirmed by an immunoblotting evaluation of PUMA protein, which allowed the exclusion of apoptosis induction by 1 mM butyrate. (**B**,**C**): In both cell lines, 1 mM butyrate (+But) significantly reduced lactate production and histone-3 lactylation. * and ** indicate a statistically significant difference in treated vs. control cells with *p* < 0.05 and 0.01, respectively, as assessed by Student’s *t*-test.

The two selected cell lines are representative of clinically relevant neoplastic diseases and were considered interesting models for the study because of their opposite features relative to lactate production and/or release. As also shown in our first experiments (see Figure 1), BxPC-3 cells display an over-activated glycolytic metabolism [21], resulting in a high-level lactate production and release. On the contrary, and despite their increased glucose uptake [22], MDA-MB-231 cells appear to release less lactate. This difference can be explained by the high pyruvate carboxylase activity of MDA-MB-231 cells [23]. Pyruvate carboxylase converts pyruvate to oxalacetate, which is rapidly consumed by the TCA cycle for sustaining cell biosynthetic reactions. For this reason, the pyruvate carboxylase reaction is essentially not reversible and, as a consequence, the generation of lactate by LDH is compromised.

This feature allowed the artificial exposure of MDA-MB-231 cells to increased lactate levels for the identification of genes responsive to the lactate-induced epigenetic changes [6]. To this aim, dose–response experiments were initially performed to identify a butyrate concentration compatible with cell viability. These experiments were performed in both cell lines maintained in their conventional medium and in MDA-MB-231 cells after a pre-conditioning exposure to 20 mM lactate for 72 h (Lac-MDA-MB-231). A total of 20 mM lactate is in line with the metabolite level detected in the microenvironment of cancer tissues [24] and, in previous experiments, it was found to induce gene expression changes [6].

Results (Figure 1A) showed that in all three cell lines no statistically significant effects on cell proliferation were observed for butyrate doses ≤ 1 mM. In MDA-MB-231 cells, 2 mM butyrate appeared to reduce cell proliferation by 20%; interestingly, this effect was found to be completely eliminated by the 20 mM lactate exposure before butyrate treatment (Lac-MDA-MB-231). This result is in agreement with previously published data suggesting a proliferative advantage awarded by lactate in cell cultures [4].

An immunoblotting evaluation of PUMA protein in both BxPC-3 and MDA-MB-231 cells exposed to 1 mM butyrate for 72 h (also shown in Figure 1A) allowed to exclude apoptosis induction and confirmed the complete tolerability of 1 mM butyrate for the two cell lines. For this reason, all subsequent analyses were performed using 1 mM butyrate, in order to investigate regulated changes occurring in viable cells and to avoid confounding effects associated with cytotoxicity.

The data of Figure 1B show that, as previously anticipated, untreated BxPC-3 cells can release in medium a lactate concentration about 2-fold higher than that released by MDA-MB-231 cells; although devoid of antiproliferative effects, 1 mM butyrate significantly reduced lactate levels in both cell lines, suggesting that this dose of metabolite can induce a metabolic rewiring characterized by glycolysis inhibition.

As is well known, one of the molecular mechanisms underlying the changes in gene expression induced by lactate is histone lactylation. Figure 1C shows that, when administered for 72 h, 1 mM butyrate caused in both cell lines a statistically significant reduction in histone-3 lactylation (≈25% and ≈60% in BxPC-3 and MDA-MB-231 cells, respectively).

This observation is in line with the idea that some lactate-induced epigenetic changes could be reversed by butyrate.

### 2.2. Butyrate and Lactate Modify in Opposite Ways Cisplatin Antineoplastic Effect

In previous studies butyrate was shown to increase the effects of different antineoplastic agents [25,26], while enhanced glycolysis and lactate production in cancer cells is a well-recognized marker of poor drug response [3,4,5,27]. Preliminarily, to explore the potential of butyrate in reversing the lactate-induced effects, we estimated the DNA damage signatures caused by cisplatin (CDDP) in cell lines exposed to the two metabolites. Cells were treated with 10 μM CDDP for 18 h, after which DNA damage was assessed by an immunoblotting evaluation of γ-H2AX, a marker of DNA double strand breaks (DSBs) [28].

**Figure 2:**
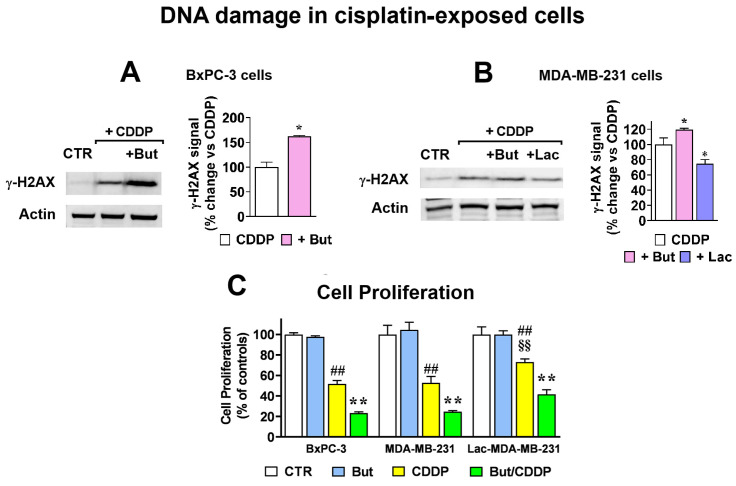
Butyrate and lactate oppositely modulate cisplatin-induced DNA damage and cytotoxicity. (**A**,**B**): 1 mM butyrate (+But) significantly increased the DNA damage signatures caused by cisplatin (CDDP) (10 μM, 18 h) in the two cell lines; (**B**) also shows that in MDA-MB-231 cells a 72 h pre-conditioning protocol with 20 mM lactate (+Lac; Lac-MDA-MB-231 cells) resulted in an opposite effect and significantly reduced CDDP-induced DNA damage. Data were analyzed by *t*-test (**A**) or one-way ANOVA followed by Dunnett’s post-test (**B**) *, *p* < 0.05 vs. cells exposed to the single CDDP treatment. (**C**) In all the three cell cultures, CDDP (5 μM, 72 h) caused a statistically significant reduction in cell proliferation, an effect further increased by butyrate (But). As shown in the bar graph, in Lac-MDA-MB-231 cells the effect of CDDP was significantly lower than that observed in the parental cell line. Data were analyzed by ANOVA followed by Tukey’s post-test: ## indicate a statistically significant difference vs. respective control cells, with *p* < 0.01; ** indicate a statistically significant difference vs. cells exposed to the single CDDP treatment, with *p* < 0.01; §§ indicate a statistically significant difference vs. MDA-MB-231 cells exposed to CDDP, with *p* < 0.01.

Figure 2A shows that in BxPC-3 cells exposed to butyrate, DNA DSBs signatures caused by CDDP were remarkably increased; a less striking but statistically significant effect was also observed in MDA-MB-231 cells (Figure 2B). Interestingly, when MDA-MB-231 cells were exposed to 20 mM lactate 72 h before CDDP treatment (+Lac; Lac-MDA-MB-231 cells), the CDDP-induced DSBs evidenced by γ-H2AX labeling were significantly reduced. The effects observed at the level of DNA damage were confirmed by the results of viability experiments. As shown in Figure 2C, in both BxPC-3 and MDA-MB-231 cells the antiproliferative effect of 5 μM CDDP was significantly increased by butyrate administration (*p* < 0.01 in both cell lines). This result was also observed in Lac-MDA-MB-231 which, in agreement with previous observations [3], proved to be less susceptible to the antineoplastic action of the drug. These results suggested that the two metabolites (butyrate and lactate) can modulate in opposite ways cell response to agents causing DNA damage and, specifically, to agents causing DSBs.

### 2.3. Butyrate and Lactate Can Differently Affect the Expression of Genes Involved in DNA Repair by Homologous Recombination

To proceed with our experiments, we referred to some previous data obtained in BxPC-3 [20] and MDA-MB-231 [6] cells.

A previous study in BxPC-3 cells suggested that homologous recombination (HR)-mediated repair is under the control of LDH activity. The reported experiments allowed us to conclude that this correlation is not linked to the enzymatic function of LDH in glycolytic flux and in ATP generation, as required for DNA repair [20].

More recently, in MDA-MB-231 cells we evidenced that exposure to 20 mM lactate leads to increased expression and release of urokinase-type plasminogen activator (uPA), a protease leading to HB-EGF release and consequent EGFR pathway activation. Interestingly, BxPC-3 is one of the cell lines for which HB-EGF-mediated EGFR pathway activation has also been described [29]. In MDA-MB-231 cells, we also showed that the 20 mM lactate exposure leads to increased gene transcription and protein level of the G-Protein Coupled Estrogen Receptor 1 (GPER1), for which a role in DNA damage repair via HR was well documented [30,31,32].

**Figure 3:**
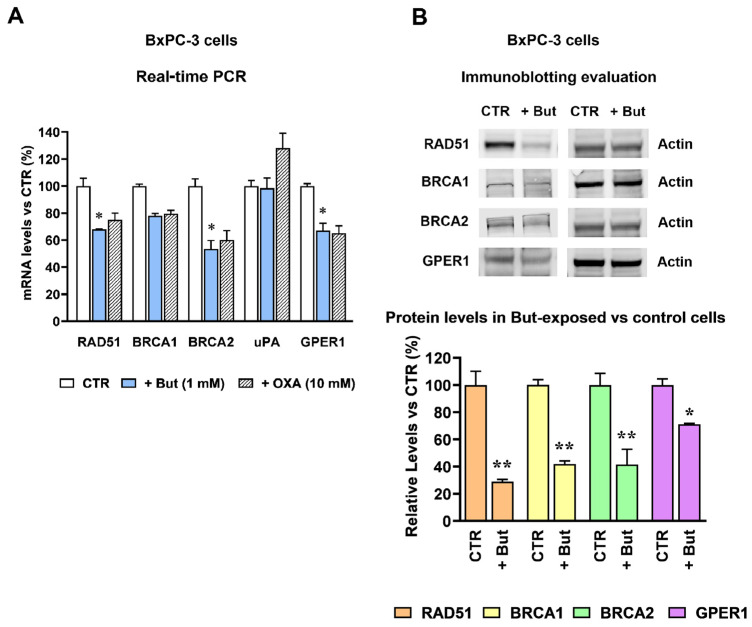
Butyrate downregulates homologous recombination-related genes in BxPC-3 cells. (**A**) Changes in gene expression caused by 1 mM butyrate (+But) and 20 mM oxamate (+OXA) in BxPC-3 cells. With the exception of BRCA1 and uPA, 1 mM butyrate exposure caused a significantly reduced gene expression. * *p* < 0.05 vs. untreated cells (CTR), as assessed by ANOVA, followed by Tukey’s post-test. No statistically significant difference was observed between the effects caused by butyrate and oxamate. (**B**) Changes in gene expression were confirmed by the immunoblotting evaluation of proteins. For each protein, the difference between untreated and butyrate-exposed cells was evaluated by *t*-test. * and ** indicate a statistically significant difference with *p* < 0.05 and 0.01, respectively.

**Figure 4:**
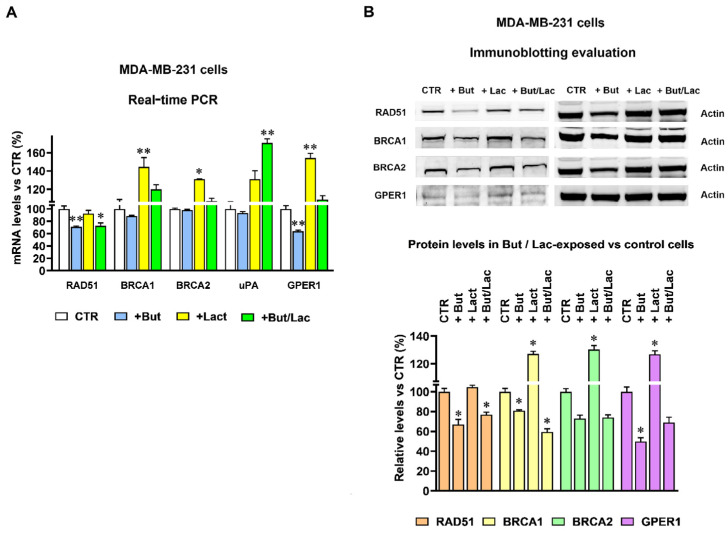
Butyrate and lactate cause opposite effects on homologous recombination-related gene expression in MDA-MB-231 cells. (**A**) Changes in gene expression caused in MDA-MB-231 cells by 1 mM butyrate (+But) and 20 mM lactate (+Lac, Lac-MDA-MB-231). Data were analyzed by ANOVA, followed by Dunnett’s post-test. * and ** indicate a statistically significant difference in treated vs. control cells with *p* < 0.05 and 0.01, respectively. (**B**) The changes in gene expression were confirmed by the immunoblotting evaluation of proteins. For each protein, the effect caused by lactate, butyrate and their association was statistically evaluated by ANOVA followed by Dunnett’s post-test. * indicates a statistically significant difference in treated vs. control cells (CTR) with *p* < 0.05.

Based on these premises, our study proceeded with the evaluation of the effects caused by butyrate and/or lactate on the expression of the three major players of HR-mediated repair (RAD51, BRCA1, BRCA2), of uPA and GPER1. For this experiment, cells were exposed to 1 mM butyrate and/or 20 mM lactate for 72 h. The obtained results are shown in Figure 3 and Figure 4 for BxPC-3 and MDA-MB-231 cells, respectively. The bar graph in Figure 3A shows that in BxPC-3 cells exposure to 1 mM butyrate reduced the expression of the HR-related genes, whereas no effect was observed concerning uPA expression. The observed effects reached the level of statistical significance with *p* < 0.05 for RAD51, BRCA2 and GPER1. Interestingly, superimposable results were observed in these cells when the butyrate treatment was replaced with a 16 h exposure to 20 mM oxamate (OXA), a well-studied LDH inhibitor [33]. In previous experiments performed on BxPC-3 cells, a similar exposure to OXA was found to markedly reduce lactate production (≈70% inhibition) without affecting cell viability [20]. These data suggest that the reduced expression of HR genes observed in butyrate-exposed BxPC-3 cells can be explained by the above-described metabolic rewiring induced by this metabolite, characterized by glycolysis inhibition and reduced lactate production (see Figure 1).

The results of Figure 3A also suggest that, contrary to what previously observed in MDA-MB-231 cells (see [6] and below), uPA expression in BxPC-3 cells is not up-regulated by lactate, since the mRNA level of this protein appeared to be even increased by OXA exposure. For this reason, uPA protein was not considered for further analysis.

The immunoblotting evaluation of the HR proteins (Figure 3B) substantially confirmed the picture observed in the RT-PCR experiment shown in Figure 3A. In this experiment, all the observed effects reached the level of statistical significance, with *p* < 0.01 (RAD51 and BRCA1) and *p* < 0.05 (BRCA2 and GPER1). It is worth noting that, with the exception of GPER1 protein, the reduction in RAD51, BRCA1 and BRCA2 protein levels appeared to be even increased when compared to the effects observed at the mRNA level. This result could be linked to the antiproliferative effect of butyrate [19], causing compromised mRNA translation of proteins mainly linked to cell cycle progression.

Similar experiments were also performed on MDA-MB-231 cells. As stated above (paragraph 2.1), the metabolic characteristics of these cells allowed us to explore the effect on gene expression caused not only by butyrate, but also by increased lactate levels and by the association of the two metabolites. The results of the RT-PCR experiments are reported in Figure 4A.

The bar graph shows that RAD51 expression was not significantly affected by lactate but, similarly to what was observed in BxPC-3 cells, butyrate exposure caused a ≈30% reduction in the RAD51 mRNA level (*p* < 0.01); lactate co-administration partially counteracted the effect of butyrate, causing a less marked reduction in RAD51 (*p* < 0.05). Figure 4A also shows that the single butyrate treatment did not significantly affect the expression of BRCA1, BRCA2 and uPA, while statistically significant mRNAs’ upregulations were caused by lactate exposure (30–37% increased expressions, with *p* < 0.05–0.01). In the case of BRCA1 and BRCA2 these effects were found to be reduced by the butyrate coadministration and, in samples receiving the combined butyrate/lactate treatment, the mRNA expression of both genes did not show statistically significant changes compared to control cells. Similarly to what was observed in BxPC-3 cells, butyrate appeared to strengthen the effect of lactate in increasing the mRNA level of uPA, and this gene was not considered for further experiments. Finally, butyrate was found to significantly reduce GPER1 expression (*p* < 0.01) and to almost completely abolish the lactate-induced up-regulation of this gene. The differences observed at the RNA levels were then confirmed by the immunoblotting evaluation. In agreement with the RT-PCR data, RAD51 protein level was not increased by lactate and was significantly reduced by butyrate exposure (*p* < 0.05) (Figure 4B). A significantly increased protein level was caused by lactate for BRCA1, BRCA2 and GPER1 (*p* < 0.05). In agreement with the RT-PCR data, these effects were counteracted by butyrate coadministration, which caused 30–45% reduced protein levels (*p* < 0.05 vs. control, untreated cells); interestingly, in samples exposed to the metabolites’ combination, the effect of butyrate appeared to prevail over the lactate-induced upregulations.

Taken together, the results obtained in BxPC-3 and MDA-MB-231 cells suggested that, because of their opposite effects on the expression genes involved in HR (BRCA1, BRCA2 and GPER1), the two considered metabolites could play a role in modulating this DNA repair mechanism.

### 2.4. The Gene Expression Changes Induced by Lactate and Butyrate Modify HR Efficiency

**Figure 5:**
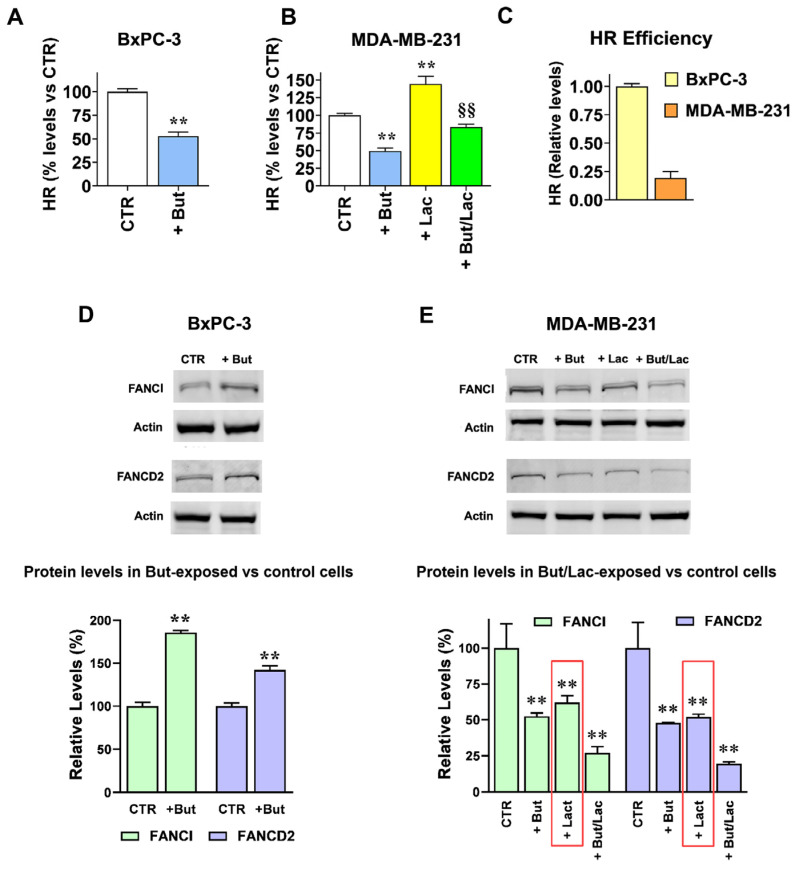
Butyrate and lactate differentially modulate homologous recombination efficiency and Fanconi anemia pathway activation. (**A**–**C**) Evaluation of HR function. In both cell lines, 1 mM butyrate treatment (+But) caused a 50% inhibition of HR function, which, on the contrary, appeared to be significantly increased by 20 mM lactate in MDA-MB-231 cells (+Lac, Lac-MDA-MB-231). Compared to BxPC-3 cells, MDA-MB-231 cells showed a 75% reduced HR function. Relative HR efficiency was calculated using the 2^−ΔΔCt^ method (fold-change) and is shown as percentage of control (control = 100%). For cross-cell line comparison (**C**), untreated BxPC-3 values were set as the calibrator (2^−ΔΔCt^ = 1). Data were analyzed by *t*-test (**A**,**C**) or ANOVA, followed by Tukey’s post-test (**B**); ** indicate a statistically significant difference in treated vs. control cells (CTR) with *p* < 0.01; §§ indicate a statistically significant difference vs. lactate-exposed cells with *p* < 0.01. (**D**,**E**) Immunoblotting evaluation of FANCI and FANCD2 proteins in butyrate and/or lactate exposed cells. In BxPC-3 cells the reduced HR function caused by 1 mM butyrate (+But) led to significantly increased FANCI and FANCD2 protein levels. In MDA-MB-231 cells the upregulated HR function caused by 20 mM lactate (+Lac, Lac-MDA-MB-231) resulted in significantly reduced FANCI and FANCD2 protein levels (red boxes). Data were analyzed by *t*-test (**D**) or ANOVA, followed by Sidak’s post-test (**E**); ** indicate a statistically significant difference in treated vs. control cells with *p* < 0.01.

To verify whether the observed changes in protein levels might affect HR efficiency, we adopted an RT-PCR based assay, requiring cell transfection with a couple of specifically designed plasmids. In the transfected cells, these plasmids operate a recombination process by exploiting the cell molecular machinery involved in HR-mediated DNA repair. This assay was repeatedly adopted by the authors in experiments aimed at assessing the power of small-molecule inhibitors of HR and proved to be a reliable tool to evaluate HR proficiency in different cell lines [34,35,36,37,38]. Results are shown in Figure 5A–C.

The bar graph in Figure 5A shows that in agreement with the results of the RT-PCR and immunoblotting experiments (Figure 3 and Figure 4), when BxPC-3 cells were exposed to butyrate the detected HR product was reduced to about 50% (*p* < 0.01); a similar result was also observed in MDA-MB-231 (Figure 5B, *p* < 0.01). In these cells, the HR-promoting effect caused by lactate was confirmed, with a 40% increased HR product; this effect was significantly reduced (*p* < 0.01), but not completely abolished, by butyrate coadministration. The RT-PCR experiment of Figure 5C also allowed a comparison between the HR proficiency of the two used cell lines. Compared to BxPC-3, MDA-MB-231 showed reduced competence in plasmids’ recombination, with a detected recombination product ≈ 75% lower than that observed in BxPC-3. This result is in line with published data [39], which explored DNA repair defects in triple negative breast cancer cell lines: compared to the other examined cells, MDA-MB-231 were found to display low proficiency in most DNA repair pathways and appeared to be the least competent in HR-mediated repair.

These results were also verified by assessing the levels of FANCI and FANCD2 proteins in the treated cells, two central components of the Fanconi anemia (FA) pathway. A substantial crosstalk between the FA and HR pathways has been evidenced and activated FA pathway also showed the potential of promoting HR-mediated repair [40]. As is well known, reduced HR function makes cells reliant on alternative and error-prone repair pathways, which triggers FA proteins’ activation. As a consequence, increased FANCI and FANCD2 levels are observed and these proteins form a heterodimer recruited to the DNA damaged site for the activation of repair processes [41].

Figure 5D shows that, in response to the butyrate-induced inhibition of HR, a statistically significant compensatory increase in FANCI and FANCD2 was observed in BxPC-3 cells. This result was not evidenced in butyrate-exposed MDA-MB-231 cells (Figure 5E); we can hypothesize that the quite low proficiency shown by these cells not only in HR but also in other DNA repair pathways [39] could also result in impaired FA/HR interaction. However, in Lac-MDA-MB-231 cells HR upregulation resulted in a statistically significant reduction in both FANCI and FANCD2 (*p* < 0.01 vs. control cells), a finding in line with the ascertained crosstalk between FA and HR pathways and with the protective effects exerted by this metabolite against DNA damage [42].

### 2.5. Butyrate and Lactate Induce Distinct Exometabolomic Changes Consistent with Their Effects on HR

**Figure 6:**
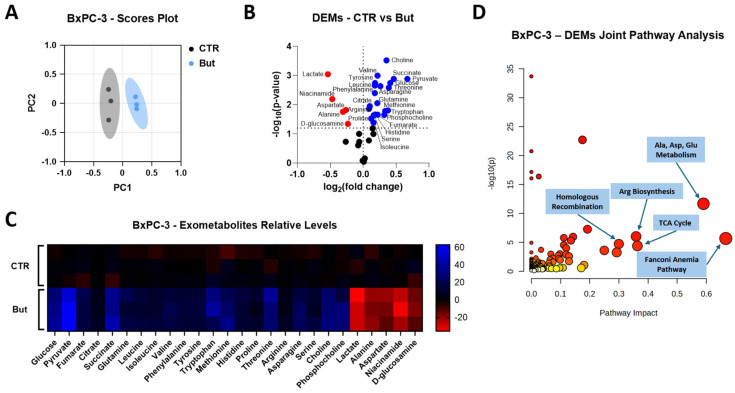
Exometabolomic profiling of BxPC-3 cells exposed to butyrate. (**A**) Principal component analysis (PCA) score plot of BxPC-3 cells cultured for 72 h in control conditions (CTR) or in the presence of 1 mM butyrate (But). Each point represents an independent biological replicate. (**B**) Volcano plot of differentially expressed metabolites (DEMs) in conditioned media from But-treated vs. CTR cells. Thresholds (dashed lines) were set at log_2_(fold change) = 0, and −log_10_(*p*-value) = 1.3 (significantly increased metabolites in red; significantly decreased metabolites in blue; non-significantly modulated metabolites in black). Statistical analysis was performed using Welch’s *t*-test (But vs. CTR) for each metabolite separately. (**C**) Heatmap of significantly altered metabolites in BxPC-3 conditioned media after 72 h treatment with But, reported as relative expression compared to CTR. (**D**) Joint pathway analysis performed in MetaboAnalyst (https://www.metaboanalyst.ca/home.xhtml, accessed on 1 December 2025 ) on BxPC-3 DEMs and differentially expressed genes (*RAD51*, *BRCA1*, *BRCA2*, *GPER1*, *uPA*, *BBC3*, *H2AX*, *FANCD2*, *FANCI*). Dot size reflects pathway impact (dot size ∝ degree centrality), and dot color encodes −log_10_(*p*-value) (dot color darkness ∝ −log_10_(*p*-value)). Enrichment analysis was performed using Fisher’s exact test, topology analysis using degree centrality, and integration using the “combine queries” option. Data are presented as mean of n = 3 biologically independent samples per condition.

Exometabolomic profiling of conditioned media has previously been exploited to monitor how cancer cells rewire nutrient uptake and secretion in response to oncogenic signals or treatments, and to integrate these extracellular signatures with gene-expression changes and DNA-damage response pathways, thereby gaining mechanistic insight into drug sensitivity and resistance [37]. Therefore, to investigate whether the distinct effects of butyrate and lactate on HR proficiency (described above) are accompanied by specific metabolic rewiring, we performed a ^1^H-NMR-based exometabolomic analysis on conditioned media collected from the same BxPC-3 and MDA-MB-231 cells exposed to butyrate and/or lactate for 72 h that underwent HR efficiency evaluation. In BxPC-3 cells, the principal component analysis (PCA) showed a clear separation between control (CTR) and butyrate-treated (But) samples (Figure 6A), indicating a substantial remodeling of extracellular metabolite profiles.

**Figure 7:**
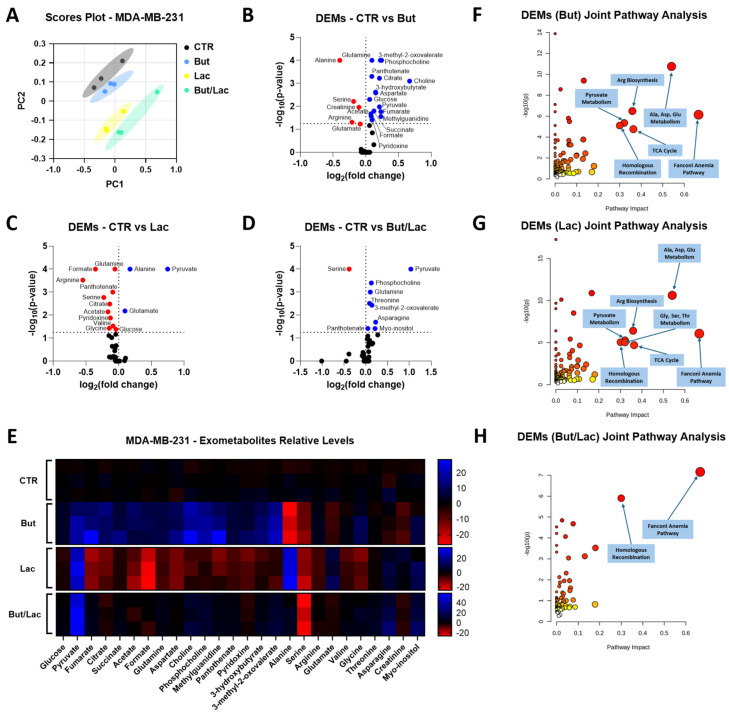
Exometabolomic changes induced by butyrate and lactate in MDA-MB-231 cells. (**A**) PCA scores plot of exometabolomic data obtained from MDA-MB-231 cells maintained in control conditions (CTR) or exposed for 72 h to 1 mM butyrate (But), 20 mM lactate (Lac) or their combination (But/Lac). (**B**–**D**) Volcano plots showing DEMs in conditioned media from But-treated (**B**), Lac-treated (**C**) and But-/Lac-treated (**D**) MDA-MB-231 cells (vs. CTR). Thresholds (dashed lines) were set at log_2_(fold change) = 0, and −log_10_(*p*-value) = 1.3 (significantly increased metabolites in red; significantly decreased metabolites in blue; non-significantly modulated metabolites in black). Statistical analysis was performed by one-way ANOVA followed by Bonferroni’s post hoc test for each metabolite separately. (**E**) Heatmap of significantly altered metabolites in MDA-MB-231 conditioned media after 72 h treatment with But, Lac or But/Lac, reported as relative expression compared to CTR. (**F**–**H**) Joint pathway analyses performed in MetaboAnalyst (https://www.metaboanalyst.ca/home.xhtml, accessed on 1 December 2025) on MDA-MB-231 DEMs and differentially expressed genes (*RAD51*, *BRCA1*, *BRCA2*, *GPER1*, *uPA*, *BBC3*, *H2AX*, *FANCD2*, *FANCI*) for But (**F**), Lac (**G**) and But/Lac (**H**) vs. CTR. Dot size reflects pathway impact (dot size ∝ degree centrality), and dot color encodes −log_10_(*p*-value) (dot color darkness ∝ −log_10_(*p*-value)). Enrichment analysis was performed using Fisher’s exact test, topology analysis using degree centrality, and integration using the “combine queries” option. Data are presented as mean of n = 3 biologically independent samples per condition.

In agreement with the glycolysis-inhibiting activity of butyrate reported in cancer cells [16,17,18,19], the volcano plot and heatmap (Figure 6B,C) showed that butyrate exposure resulted in a significant increase in glucose in the medium, together with reduced lactate and alanine levels. Concurrently, pyruvate and several TCA intermediates (citrate, succinate and fumarate) appeared to accumulate extracellularly. Most amino acids, including branched-chain and aromatic amino acids, glutamine, methionine, histidine, proline, threonine, arginine, asparagine and serine, as well as choline and phosphocholine, were also increased in conditioned media from butyrate-treated cells, whereas aspartate, glutamate, niacinamide and D-glucosamine were significantly decreased. Overall, these data suggest that in BxPC-3 cells butyrate induces a metabolic rewiring characterized by reduced glucose consumption and impaired utilization of TCA intermediates, several amino acids and other metabolites required to sustain nucleotide synthesis and efficient DNA repair [43]. To explore the functional meaning of these alterations, joint pathway analysis was performed in the online platform MetaboAnalyst by integrating the differentially expressed metabolites (DEMs) with the expression changes in the genes investigated in the same experimental conditions (i.e., *RAD51*, *BRCA1*, *BRCA2*, *GPER1*, *uPA*, *BBC3*, *H2AX*, *FANCD2* and *FANCI*). This analysis revealed that HR and FA pathways, together with the TCA cycle, arginine biosynthesis and alanine, aspartate and glutamate metabolism, were the pathways with the highest topological impact (Figure 6D). These results are consistent with the tight functional interplay between FA and HR in the repair of replication-associated lesions [40,41] as well as with the recognized requirement for aspartate, glutamine and other TCA-linked metabolites to sustain de novo nucleotide synthesis and DNA repair [43]. Collectively, these findings support the notion that the butyrate-induced inhibition of HR in BxPC-3 cells occurs in a metabolically unfavorable context for high-fidelity DNA repair [44,45]. A similar approach was applied to MDA-MB-231 cells, where the metabolic effects of both butyrate and lactate, alone or in combination, were investigated. The PCA score plot (Figure 7A) evidenced four distinct clusters corresponding to control (CTR), butyrate- (But), lactate- (Lac) and butyrate/lactate coadministration-treated (But/Lac) samples, indicating that each treatment was associated with a specific exometabolomic profile.

In this analysis, only metabolites showing statistically significant changes were reported and discussed in the Results, whereas the heatmap was designed to display all detected metabolites across the different treatments in order to facilitate visual comparison and to highlight the overall trend toward normalization observed in the combined butyrate/lactate condition. In agreement with the results obtained in BxPC-3 cells, butyrate increased extracellular glucose, pyruvate and TCA intermediates (citrate, succinate, fumarate), together with glutamine, choline, phosphocholine, pantothenic acid and pyridoxine (Figure 7B,E). The levels of acetate, 3-hydroxybutyrate and 3-methyl-2-oxovaleric acid were also increased, while alanine, serine, arginine, glutamate and creatinine were reduced, and methylguanidine appeared significantly increased, suggesting enhanced oxidative stress. These data indicate that, also in MDA-MB-231 cells, butyrate induces a condition of mitochondrial and redox stress, accompanied by reduced amino acid uptake and accumulation of incompletely oxidized substrates [16,17,18,19], also in agreement with the dual role of butyrate as an HDAC inhibitor and metabolic modulator [46]. On the contrary, lactate exposure caused an almost specular effect. In line with the role of lactate as an oxidative substrate and signaling molecule in cancer cells [3,4,24], Lac-treated MDA-MB-231 cells displayed increased pyruvate, alanine and glutamate in the medium, together with reduced glucose, glutamine, choline, phosphocholine, pantothenate, TCA intermediates, acetate, 3-hydroxybutyrate, 3-methyl-2-oxovalerate, methylguanidine, formate, pyridoxine, serine, arginine, valine and glycine (Figure 7C,E). This pattern is compatible with efficient lactate uptake and oxidation, activation of pyruvate–alanine cycling, reduced reliance on branched-chain amino acids for anaplerosis and lower oxidative stress. These changes are consistent with the role of lactate as an oxidative fuel in breast cancer cells [47,48] and with recent evidence that lactate and lactylation can promote the activity of HR proteins such as MRE11 in response to DNA damage [42,49]. Interestingly, in the But/Lac condition many metabolites showed intermediate or partially normalized values compared to those observed in the single treatments (Figure 7D,E). Glutamine, phosphocholine, pantothenic acid, pyruvate, threonine, myo-inositol, asparagine and 3-methyl-2-oxovalerate were significantly increased, while serine was reduced in the medium. Joint pathway analysis, integrating metabolite changes with the expression of the same genes previously investigated, identified pyruvate metabolism, amino acid metabolism (alanine, aspartate and glutamate; glycine, serine and threonine; arginine biosynthesis), TCA cycle, HR and FA as the most impacted pathways for butyrate and lactate single treatments (Figure 7F,G). Notably, in the combined But/Lac condition only HR and FA pathway remained as high-impact nodes (Figure 7H). Given the close functional interplay between HR and FA pathways in the repair of replication-associated lesions and interstrand crosslinks [40,50], these data suggest that lactate can mitigate several butyrate-induced metabolic defects but does not fully restore DNA repair pathway homeostasis at the transcriptional/protein level at the investigated timepoint. Together with the HR assays, these exometabolomic results support a model in which butyrate drives a metabolically and epigenetically unfavorable state for HR, whereas lactate promotes an oxidative, HR-permissive state, and the combination yields an intermediate phenotype in which DNA repair pathways remain the major functional impacted aspect.

### 2.6. The HR-Modulatory Effects of Butyrate and Lactate Modify Cell Response to PARP and RAD52 Inhibitors

**Figure 8:**
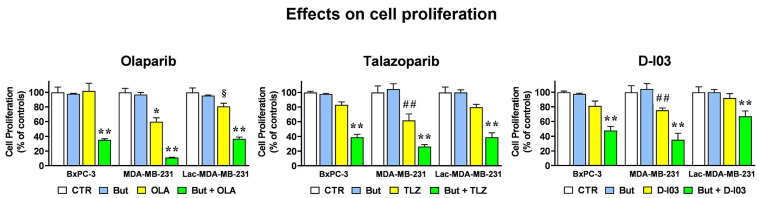
Butyrate enhances sensitivity to PARP and RAD52 inhibitors and counteracts lactate-associated resistance. Effects caused by the PARPi Olaparib, 10 μM (OLA), Talazoparib, 2 μM (TLZ), and by the RAD52 inhibitor D-I03, 15 μM, on the proliferation of cells exposed to 1 mM butyrate (But) and/or 20 mM lactate (Lac-MDA-MB-231). Data were analyzed by ANOVA, followed by Tukeys’ post-test. * Indicates a statistically significant difference in treated vs. control MDA-MB-231 cells (CTR) with *p* < 0.05. ## Indicate a statistically significant difference in treated vs. control MDA-MB-231 cells with *p* < 0.01. ** Indicate a statistically significant difference vs. the single PARPi or D-I03 treatment, with *p* < 0.01. § Indicates a statistically significant difference between the effect caused by Olaparib in MDA-MB-231 cells vs. Lac-MDA-MB-231 cells.

As well documented, reduced HR efficiency is a feature predicting susceptibility of cancer cells to PARP inhibitors (PARPi) [51] and can also make cancer cells responsive to RAD52 inhibitors [52]. RAD52 is involved in DSBs repair [53] and was found to be essential for the viability of BRCA1 and BRCA2 deficient cells, but not for that of normal cells, which suggested this protein as an attractive therapeutic target for the hereditary pancreatic, breast and ovarian cancers syndromes. To conclude our study, we then verified whether the HR-modulatory effect exerted by lactate and butyrate in BxPC-3 and MDA-MB-231 cells could affect the cell response to olaparib and talazoparib (two widely studied PARPi) and to D-I03, a recently developed and selective RAD52 inhibitor [54]. Results are shown in Figure 8; for each inhibitor, the bar graphs show the effects caused by a 72 h co-treatment with 1 mM butyrate in BxPC-3, MDA-MB-231 and lactate-exposed MDA-MB-231 (Lac-MDA-MB-231) cells. The single administration of butyrate did not affect cell proliferation in the three cell lines. In BxPC-3 cells, the single treatments with the PARPi(s) and with D-I03 did not affect cell proliferation, which on the contrary was significantly reduced when each of the three inhibitors was administered in association with butyrate (*p* < 0.01 for all the three compounds). As expected, and in agreement with the low proficiency in DNA repair shown for these cells [39], MDA-MB-231 cells showed higher response to the three tested inhibitors, with a statistically significant difference vs. the untreated cells. Lactate exposure was found to reduce the effect of the three inhibitors (Lac-MDA-MB-231 cells), a result reaching the level of statistical significance in the case of olaparib.

The lower response of the Lac-MDA-MB-231 cells to the studied inhibitors is in complete agreement with the lactate-induced upregulation of HR process shown in Figure 4 and Figure 5B and is in line with the effect exerted by this metabolite in increasing drug resistance [3,4]. However, and as also shown by the data of Figure 4B and Figure 5C,E, when MDA-MB-231 cells were exposed to both metabolites the effect of butyrate prevailed over the lactate-induced drug resistance: in Lac-MDA-MB-231 cells the effects of all the three tested inhibitors were significantly increased by butyrate, with a remarkable difference observed in the case of the two PARPi.

Overall, the results of the viability experiments described in Figure 8 are in agreement with all the results shown in previous figures, which fully supports the hypothesis of a butyrate-/lactate-mediated control on HR function.

## 3. Discussion

LDH expression has been recognized as one of the major determinants affecting tumor prognosis [55]. A recent meta-analysis revealed that high LDH activity was positively correlated with the presence of stemness scores and was able to predict poor chemotherapy response in multiple human cancers [56,57]. As suggested by several studies, in tumor cells part of the deleterious effects linked to the increased LDH expression and activity can be ascribed to lactate, the product of LDH reaction [3,4,5,6]. By causing histone hyperacetylation or lactylation, this metabolite was found to promote stem properties, infiltrative growth and drug resistance [8,9]. The data reported in this manuscript suggest that at least one of the lactate-induced deleterious effects (drug resistance) could be effectively dampened by a second metabolite, butyrate. In agreement with this hypothesis, in butyrate exposed cells our experiments showed reduced lactate production (Figure 1B) and reduced histone-3 lactylation, a lactate-specific mechanism affecting gene expression [8].

We hypothesized that the lactate-induced cancer promoting effects could be restrained by butyrate for its potential of enhancing mitochondrial function and promoting oxidative metabolism reactions [17]. The biological effects exerted by both metabolites are usually mediated by HDAC inhibition. Specifically, lactate was found to be linked to Class I HDAC (HDAC1, 2, 3) [58]. Interestingly, beside operating histone acetylation, these enzymes were also found to catalyze the addition of lactate residues to histones and other proteins. High level of protein lactylation seems to be linked to the high intracellular lactate concentration associated with the Warburg effect, a condition which also leads to the inhibition of the deacetylase activity [58].

Broader action on HDACs was instead described for butyrate, since this metabolite was shown to inhibit several Class I and II enzymes; its function is usually associated with cell cycle arrest, cell differentiation and/or apoptosis [58]. Based on these premises, the counteracting effects observed in our experiments could be explained by a different specificity of butyrate and lactate for distinct HDAC enzymes.

The exometabolomic analyses performed in the two cell lines further support this hypothesis and provide a mechanistic link between the epigenetic effects of the metabolites and the observed modulation of HR. In BxPC-3 cells, butyrate exposure caused a marked reduction in glycolytic flux, as indicated by increased extracellular glucose and reduced lactate, together with accumulation of TCA intermediates and decreased uptake of several amino acids. In particular, the reduced levels of aspartate, glutamate and niacinamide in conditioned media are compatible with a limitation of nucleotide synthesis and NAD^+^ turnover, two conditions known to compromise DNA replication and repair [43]. In MDA-MB-231 cells, butyrate induced a similar metabolic stress signature, with increased TCA intermediates, ketone and acetate overflow and accumulation of the oxidative stress marker methylguanidine, whereas lactate produced a specular pattern characterized by increased pyruvate and alanine release and reduced TCA and ROS-related metabolites. Joint pathway analyses integrating metabolite changes with the expression of RAD51, BRCA1, BRCA2, FANCD2 and FANCI consistently highlighted HR and the FA pathway among the most impacted pathways, and in the combined butyrate/lactate condition these pathways remained predominant despite partial normalization of other metabolic parameters. These findings reinforce the idea that the opposite effects of lactate and butyrate are mediated not only by their direct epigenetic actions on chromatin [8,9,13,58], but also by a profound remodeling of cellular metabolism that selectively favors or impairs HR-mediated DNA repair.

It should be noted that the experiments reported in this study were conducted using two human neoplastic cell lines selected as representative models of pancreatic ductal adenocarcinoma (BxPC-3) and triple-negative breast cancer (MDA-MB-231) and on the basis of their distinct metabolic features (particularly regarding lactate) [5,6,20,21] and DNA repair proficiency [20,39]. While these models allowed us to explore the interplay between lactate and butyrate in tumor cells with different metabolic wiring, it should be acknowledged that tumor heterogeneity may result in variable responses to these metabolites. Therefore, the conclusions drawn from the present work should be interpreted within the context of the employed experimental models. Nevertheless, the coherent modulation of HR and drug response observed in both cell lines supports the concept that the balance between lactate- and butyrate-driven metabolic states can influence DNA repair capacity.

Together with other short-chain fatty acids, butyrate is obtained from the fermentation of complex substrates, operated by different bacterial species in human intestine [59]. Microbiome-derived butyrate is rapidly taken up by colonocytes, but also enters the circulation, reaching the liver and different peripheral tissues [60]. In colonocytes, butyrate primarily functions as an energy substrate [61] and the metabolite fraction that is not utilized by these cells is transported by the portal vein to the liver [60]. Hepatocytes were shown to exploit butyrate for ketogenesis and triacylglycerol synthesis [62]. In both colon and liver, this metabolite was also found to exert anti-inflammatory and antitumor effects, which can be ascribed to its immunomodulatory properties [63]. Because of the high butyrate concentration detected in human colon lumen, intestine and liver are particularly exposed to the health effects of this metabolite; in the systemic circulation the level of butyrate was found to be ≤15 μM, about 2% of that measured in the colon [60]. For this reason and to benefit from the healthy properties of the metabolite, oral supplementation of butyrate was proposed. However, this procedure appeared to cause only transiently increased blood levels of the metabolite [64,65]. On the other hand, and despite this discouraging finding, ever increasing evidence indicates a protective role for butyrate also in pathologic conditions affecting sites different from liver and intestine.

Clinical observations suggested that the epigenetic changes induced by butyrate can protectively modulate immune response: bacterial butyrate was found to protect children from developing atopy [66]. Furthermore, higher fecal levels of this metabolite were found to be associated with a reduced risk to develop asthma and food allergy [67].

Other studies recognized butyrate as a modulator of neurological health, linked to its interaction with the gut–brain axis [68]. Clinical evidence indicated that butyrate can alleviate neurological disorders, such as Alzheimer’s and Parkinson’s diseases and autism spectrum disorders [69,70,71]. These effects were found to be linked to increased histone acetylation, causing reduced neuroinflammation and enhanced neurotransmitters’ modulation [66]. The anti-inflammatory properties of this metabolite could probably be involved also in protective effects against atherosclerosis progression: in cultured endothelial cells, butyrate was found to reduce VCAM1 expression [72] and pro-inflammatory cytokines production [73]. Furthermore, reduced aortic atherosclerosis and improved plaque stability was observed in the ApoE^−/−^ mice model after an oral supplementation with butyrate [74].

Although health-beneficial effects of butyrate have already been described, to our knowledge the experiments reported in this manuscript show for the first time a counteracting effect on gene regulation induced by lactate and butyrate, two metabolites affecting cancer cell biological properties. Since poor drug response is one of the best characterized and most clinically relevant consequences of the increased lactate production of cancer cells, our experiments explored the effects caused by the two metabolites on DNA repair. Specifically, opposing effects of lactate and butyrate on HR-mediated repair were evidenced. In addition to highlighting a counteracting effect caused by the two metabolites, the butyrate-induced HR inhibition observed in our experiments also suggested supplementation with this metabolite as a possible way to extend the use of PARPi to the cancer forms poorly responsive to these inhibitors; this could be an interesting therapeutic attempt, also considering the good tolerability of this anticancer treatment.

Besides fostering drug resistance, lactate was also described as a promoter of immune-inflammatory responses [55]. Since immune-modulatory and anti-inflammatory properties have been shown for butyrate [63], our results also suggest that the counteracting effect between these two metabolites could not be limited to DNA repair but could also be relevant in the management of these critical issues, often affecting the success of anticancer treatments.

## 4. Materials and Methods

### 4.1. Cell Lines and Treatments

MDA-MB-231 and BxPC_3_ cells (ATCC, Manassas, VA, USA) were grown in low-glucose (1 g/L) Dulbecco’s minimal essential medium (DMEM) (31885-023, Thermo Fisher Scientific, Waltham, MA, USA) and Roswell Park Memorial Institute (RPMI) (R0883, Merck, Darmstadt, Germany) respectively. Media were supplemented with 100 U/mL penicillin/streptomycin (P0871, Merck, Darmstadt, Germany), 2 mM glutamine (G7513, Merck, Darmstadt, Germany) and 10% FBS (ECS5000L, Euroclone, Milan, Italy). L-lactate (439220100, Thermo Fisher Scientific, Waltham, MA, USA) was dissolved in PBS (D8537, Merck, Darmstadt, Germany) and added to DMEM medium at a final concentration of 20 mM. MDA-MB-231 cells were exposed to 20 mM lactate as a pre-conditioning protocol, 72 h before experiments (Lac-MDA-MB-231). Sodium butyrate (B-5887, Merck, Darmstadt, Germany) was obtained in lyophilized form; it was dissolved in ultra-pure water (W4502, Merck, Darmstadt, Germany) and stored at −20 °C. For butyrate-including experiments, all cell cultures were exposed to 1 mM dose of the metabolite for 72 h. This treatment was performed as a pre-conditioning protocol in all experiments, with the exception of those shown in Figure 1A,C and Figure 8 (cell proliferation experiments at 72 h), for which cell cultures were directly exposed to the metabolite without a pre-conditioning phase. Cisplatin (CDDP, HY-17394, Med Chem Express, Monmouth Junction, NJ, USA) was obtained in lyophilized form; it was dissolved in 0.9% NaCl and stored at −20 °C. OXA (O2751, Merck, Darmstadt, Germany) was obtained in lyophilized form and was dissolved in RPMI medium at a final concentration of 20 mM. Olaparib (Selleck Chemicals, Houston, TX, USA) was obtained in lyophilized form and was dissolved in DMSO (C6164, Merck, Darmstadt, Germany); all cell cultures were exposed to 10 μM olaparib for 72 h. Talazoparib (S7048, Selleck Chemicals, Houston, TX, USA) was obtained in lyophilized form; it was dissolved in DMSO and used at a final concentration of 2 μM (72 h) in all cell cultures. D-I03 (Merck, Darmstadt, Germany) was obtained in lyophilized form; it was dissolved in DMSO and used at a final concentration of 15 μM (72 h) in all cell cultures.

### 4.2. Cell Proliferation Experiments

Cell proliferation was assessed by crystal violet staining. Crystal violet (CV, C0775, Merck, Darmstadt, Germany) was obtained in lyophilized form and dissolved in distilled water. MDA-MB-231, Lac-MDA-MB-231 and BxPC-3 cells were exposed to butyrate and/or to the selected inhibitors at the indicated doses and times. In these experiments, Lac-MDA-MB-231 received the 20 mM lactate supplementation also during the 72 h treatments (Figure 1A, Figure 2C and Figure 8), to ensure the stability of gene expression changes induced by lactate. Cells (4 × 10^3^/well) were seeded in 96-multiwell plates and let to adhere overnight. They were then exposed to the selected inhibitors for 72 h. At the end of treatment, medium was removed and cells were fixed with 1% glutaraldehyde (G6257, Merck, Darmstadt, Germany) for 20 min. Fixed cells were then stained with a 0.01% CV solution for 30 min. After staining, they were washed with PBS (D8537, Merck, Darmstadt, Germany) for three times and CV was solubilized by shaking in 70% ice-cold ethanol for 30 min at room temperature. Absorbance was measured at 570 nm using a Multiskan EX plate reader (Thermo Fisher Scientific). For each experiment and treatment, changes in cell number over the 72-h period were calculated. In cell proliferation experiments, untreated Lac-MDA-MB-231 were used as a control reference to assess the effect of the tested inhibitors in lactate-exposed cells.

### 4.3. Evaluation of Lactate Level

MDA-MB-231 and BxPC-3 cells were treated with 1 mM butyrate for 72 h. Following treatment, cells were seeded in triplicate into 24-well plates (2 × 10^5^ cells/well) and allowed to adhere overnight. The culture medium was then replaced with 300 μL Krebs–Ringer buffer per well. The concentration of lactate released into the buffer was measured after 3 h of incubation at 37 °C, following the procedure described in [5].

### 4.4. Real-Time PCR

Real-time PCR (RT-PCR) was performed on BxPC-3 cells and on control and lactate-exposed MDA-MB-231 cells (Lac-MDA-MB-231) treated with 1 mM butyrate for 72 h. BxPC-3 cells were also treated with 20 mM OXA for 16 h. Exponentially growing cells cultured in T25 flasks were used for RNA extraction, which was performed with an RNA isolation kit (83912, Merck, Darmstadt, Germany). RNA quantity and purity were assessed spectrophotometrically using an ONDA Nano Genius photometer (OPTO-LAB Instruments, Modena, Italy). Complementary DNA (cDNA) was synthesized from total RNA using the Revert Aid First Strand cDNA Synthesis Kit (K1691, lot 00291984, Thermo Fisher Scientific) following these steps: denaturation at 65 °C for 5 min, primer annealing at 25 °C for 5 min, reverse transcription for 1 h at 42 °C and enzyme inactivation for 5 min at 70 °C. Quantitative RT–PCR was performed using 20 ng of cDNA, Sso-Advanced™ Universal SYBR^®^ Green Supermix (1725271, lot 64545727; Bio-Rad, Hercules, CA, USA), and specific primer mixtures. All primers used in the PCR experiments were predesigned (KiCqStart^®^, Merck). The used reference genes were CYP33, RPLP0, and B2M. The list of the oligonucleotide primer pairs (Merck, Darmstadt, Germany) is shown in Table 1. For all genes, primer annealing and extension were performed at 60 °C, and amplification was carried out on a CFX96™ Real-Time PCR System (Bio-Rad, Hercules, CA, USA) using the following program: initial denaturation at 95 °C for 30 s, followed by 40 cycles of 95 °C for 15 s and 60 °C for 30 s. Relative gene expression levels were calculated using the 2^−ΔΔCt^ method. In the RT-PCR assays, results obtained in lactate exposed cells (Lac-MDA-MB-231) were evaluated by using untreated MDA-MB-231 cells as the reference control of experiments. This procedure was followed in order to assess the gene expression changes induced by lactate.

### 4.5. Immunoblotting Experiments

These experiments were performed on control and lactate-exposed MDA-MB-231 and on BxPC-3 cells. Cultures from T25 flasks at approximately 80% confluence were harvested and lysed in 50 µL RIPA buffer supplemented with protease and phosphatase inhibitors (cOmplete™, 04693116001, Merck; Halt™, 78420, Thermo Fisher Scientific). For each sample, proteins were quantified using the Bradford assay (B6916, Merck) and 70 µg samples were separated by electrophoresis on 4–12% precast polyacrylamide gels (Bolt™, 04120, Thermo Fisher Scientific), under a constant voltage of 170 V. Proteins were subsequently transferred onto low-fluorescence Hybond™ PVDF membranes (10600060, lot A30730600; Cytiva, GE Healthcare, Chicago, IL, USA) using the Bolt™ transfer system at 60 mA for 16 h. Membranes were then blocked for 1 h with 5% BSA (A9418, Merck) dissolved in TBS-Tween and then incubated with the appropriate primary antibody. Actin served as the loading control in all experiments. The antibodies (primary and secondary) used for immunoblotting experiments are listed in Table 2. Membrane fluorescence was detected using Chemi-Doc MP Imaging System (Bio-Rad, Hercules, CA, USA), and band intensities were quantified with the ImageJ software (version 1.53a). In the immunoblotting assays, results obtained in lactate-exposed cells (Lac-MDA-MB-231) were evaluated by using untreated MDA-MB-231 cells as the reference control of experiments. This procedure was followed in order to assess the protein level changes induced by lactate.

### 4.6. Homologous Recombination Assay

Homologous recombination (HR) activity was evaluated using a commercially available assay kit (Norgen, Thorold, ON, Canada), as previously described [75]. MDA-MB-231, Lac-MDA-MB-231 and BxPC-3 cells were treated with 1 mM butyrate for 72 h. Following treatment, cells were seeded (2 × 10^5^ cells per well) into 24-well plates and allowed to adhere overnight. Co-transfection with the two reporter plasmids was carried out using Lipofectamine™ 2000 (11668030, Invitrogen, Thermo Fisher Scientific, Waltham, MA, USA), according to the manufacturer’s instructions. Following transfection, cells were washed with PBS, collected, and genomic DNA was extracted using the Illustra Tissue and Cell Genomic Prep Mini Spin Kit (GE Healthcare). DNA concentration and purity were determined with an ONDA Nano Genius photometer (OPTO-LAB Instruments, Modena, Italy). HR efficiency was quantified by real-time PCR using 25 ng of template DNA, the primer mixtures supplied with the assay kit, and the manufacturer’s recommended cycling conditions. Relative HR efficiency was calculated using the 2^−ΔΔCt^ method, by comparing the ratio (recombination product/backbone plasmid) in treated samples versus untreated controls.

### 4.7. NMR Exometabolomic Analysis

*Sample preparation*. Metabolomic studies on cell culture media to investigate treatment-induced changes on mitochondrial function and oxidative metabolism in BxPC-3 cells were performed as described in [37]. Culture media of the same cells used for the homologous recombination assay were collected right before DNA extraction, immediately frozen in liquid nitrogen and stored at −80 °C. Right before NMR analysis, culture media of cells exposed to the different treatments were thawed on ice, centrifuged at 20,000× *g* 4 °C for 15 min. A total of 400 μL of each sample was diluted with 100 μL of a prepared NMR buffer for a final concentration of 150 mM buffer phosphate pH 7.4, 1 mM 2,2′,3,3′-deuterotrimethylsilylproprionic acid (TSP) as chemical shift reference, 0.04% sodium azide, and 20% D_2_O (for the lock signal) into a 5 mm NMR tube.

*NMR analysis*. All the NMR experiments were recorded with a UltraShield Plus FT-NMR 600 MHz AVANCE NEO (Bruker Daltonics, Bremen, Germany) equipped with a Cryoprobe™ QCI ^1^H/^19^F–^13^C/^15^N–D (Bruker Daltonics) with a SampleJet™ autosampler (Bruker Daltonics) with temperature control. For each sample, the probe was automatically locked, tuned, matched, and shimmed. Before measurement, the samples were kept for 5 min inside the NMR probe head for temperature equilibration at 298 K. Two NMR spectra were recorded for each sample: a monodimensional (1D) ^1^H NMR spectrum with a standard pulse sequence water suppression (noesygppr1d, Bruker), with 128 scans, 64,000 data points, a spectral width of 30 ppm, an acquisition time of 1.835 s, a relaxation delay of 4 s, and a mixing time of 100 ms and a 1D ^1^H spin-echo Carr-Purcell-Meiboom-Gill sequence (cpmgpr1d, Bruker) to suppress large NMR signals arising from high molecular weight molecules (i.e., serum proteins). In the cpmg experiments, the total echo time was 38.4 ms consisting of 128 repetitions with a τ time of 300 μs and a 180° pulse of approximately 36 μs. Each spectrum was recorded with a total of 128 scans, 64,000 data points, a repetition time of 4 s, and an acquisition time of 1.835 s. The free induction decay was multiplied with an exponential window function with 0.3 Hz line broadening prior to Fourier transformation. All the ^1^H NMR chemical shifts are referenced to the TSP signal.

*Metabolomic analysis*. Obtained spectra were analyzed MestReNova (version 15.01) Chemometrics package (Mestrelab Research S.L., Santiago de Compostela, Spain) from spectra processing, bucketing, and normalization to statistical principal component analysis (PCA). For bucketing, a width of 0.04 ppm was used, and the samples were normalized based on the total intensity (each bucket integration is divided by the integration of the total spectrum). The significant NMR buckets, resulted from MetReNova, were assigned by Assure2.2 Bruker program, the Human Metabolome Database (https://hmdb.ca/, accessed on 25 September 2025) and Chenomx Profiler (Chenomx NMR suite 8.5 evaluation). The identification and quantification of the different metabolites was obtained automatically by Assure 2.2, using an external standard (10 mM dimethyl malonic acid), and the PULCON method. The consumption or the production/release of each metabolite was calculated by comparing their amounts with those in the procedural blank (medium incubated in the same experimental conditions but without cells). Only significant (*p* < 0.05 as reference value, Welch’s *t*-test or one-way ANOVA followed by Bonferroni’s post hoc test) consumption/release values were included in the respective figure.

### 4.8. Statistical Analyses

Results were obtained from at least two independent experiments, performed with triplicate samples. Data were analyzed using the GraphPad Prism 10 softwares. For each experiment, the adopted statistical evaluation is described in the corresponding figure caption of Section 2. Data were expressed as mean values ± SE; the significance level was set at *p* < 0.05. For the exometabolomic analysis, thresholds for statistical significance were set at −log_10_(*p*-value) = 1.3 (i.e., *p*-value = 0.05) and log_2_(fold change) = 0 (i.e., fold change = 1). Joint pathway analysis was performed using the online platform MetaboAnalyst (https://www.metaboanalyst.ca/home.xhtml, accessed on 1 December 2025), selecting the “Pathway Analysis” function and the following parameters: Fisher’s exact test for enrichment analysis, degree centrality for topology measure, combined queries for integration method.

## Figures and Tables

**Table 1:** Details of oligonucleotide primer pairs used in the RT-PCR experiments.

Gene	ID	Forward (5′-3′)	Reverse (5′-3′)
*RAD51*	NM_001164269	CAAATGCAGATACTTCAGTGGAA	TCCAGCTTCTTCCAATTTCTTCA
*BRCA1*	NM_007294	TATCCGCTGCTTTGTCCTCA	TGCAGGAAACCAGTCTCAGT
*BRCA2*	NM_000059	GAGAGTTCCCAGGCCAGTA	ACTGGAAAGGTTAAGCGTCA
*uPA*	NM_001145031	GAAAACCTCATCCTACACAAG	ATTCTCTTTTCCAAAGCCAG
*GPER1*	NM_001039966	TTCCGCGAGAAGATGACCATCC	TAGTACCGCTCGTGCAGGTTGA
*CYP33A (PPIE)*	NM_006112	GCTGCCTGTGCACTCATGAA	CAGTGCCATTGTGGTTTGTGA
*RPLP0*	NR_002775	CAGATTGGCTACCCAACTGTT	GGCCAGGACTCGTTTGTACC
*B2M*	NM_004048	CATTCCTGAAGCTGACAGCATTC	TGCTGGATGACGTGAGTA

**Table 2:** Details of antibodies applied for protein detection.

Experiments	Antibody	Host Species	Catalog No.	Producer	Dilution	Time
IB, primary	PUMA α/β	Mouse	Sc-374223	Santa Cruz ^(a)^	1:500	16 h (4 °C)
	Lactyl-H3	Rabbit	A21214	ABClonal ^(b)^	1:3000	1 h
	RAD51	Rabbit	70-012	Bio Academia ^(c)^	1:1000	1 h
	BRCA1	Rabbit	22362-1-AP	Proteintech ^(d)^	1:2000	16 h (4 °C)
	BRCA2	Rabbit	9012	Cell Signaling ^(e)^	1:1000	1 h
	BRCA2 *	Rabbit	A2435	ABClonal	1:750	16 h (4 °C)
	GPER1	Rabbit	A10217	ABClonal	1:1000	1 h
	* γ-H2AX *	Rabbit	Ab11174	Abcam ^(f)^	1:2000	1 h
	* FANCI *	Rabbit	Ab245219	Abcam	1:1000	16 h (4 °C)
	* FAND2 *	Rabbit	Ab178705	Abcam	1:2500	16 h (4 °C)
	Actin	Rabbit	A2066	Merck	1:1000	2 h
IB, secondary	Rabbit IgG Cy5-labeled	Goat	111-175-144	Jackson Immuno Research ^(g)^	1:2500	1 h
	Mouse IgG Alexa-Fluor 647-labeled	Donkey	715-605-151	Jackson Immuno Research	1:1000	1 h

(a): Santa Cruz Biotechnology Inc., Dallas, TX, USA. (b): AB clonal: German GmbH, Düsseldorf, Germany. (c): Bio academia: Osaka University, Osaka, Japan. (d): Proteintech: Rosemont, IL, USA. (e): Cell Signaling: Danvers, MA, USA. (f): Abcam: Cambridge, UK. (g): Jackson Immuno Research: Ely, Cambridgeshire CB7 4 EX, UK. * This antibody was used in MDA-MB-231 cells.

## Data Availability

The original contributions presented in this study are entirely included in the article. Further inquiries can be directed to the corresponding author.

## Abbreviations

The following abbreviations are used in this manuscript:

But	Butyrate
CDDP	Cisplatin
DSB	Double Strand Breaks
FA	Fanconi Anemia
HDAC	Histone Deacetylase
HR	Homologous Recombination
Lac	Lactate
LDH	Lactate Dehydrogenase
OXA	Oxamate
PARPi	Poly-ADP-Ribose Polymerase Inhibitor
RT-PCR	Real Time PCR
